# Medicalized Female Genital Mutilation/Cutting: Contentious Practices and Persistent Debates

**DOI:** 10.1007/s11930-018-0140-y

**Published:** 2018-02-21

**Authors:** Samuel Kimani, Bettina Shell-Duncan

**Affiliations:** 1 Africa Coordinating Centre for Abandonment of FGM/C (ACCAF), University of Nairobi, Nairobi, Kenya; 2 Departments of Anthropology and Global Health, University of Washington, Box 353100, Seattle, WA 98105-3100 USA

**Keywords:** Female genital mutilation, Female genital cutting, Medicalization, Harm reduction, Health care providers

## Abstract

**Purpose of Review:**

Female genital cutting/mutilation (FGM/C) performed by health care professionals (medicalization) and reduced severity of cutting have been advanced as strategies for minimizing health risks, sparking acrimonious ongoing debates. This study summarizes key debates and critically assesses supporting evidence.

**Recent Findings:**

While medicalization is concentrated in Africa, health professionals worldwide have faced requests to perform FGM/C. Whether medicalization is hindering the decline of FGM/C is unclear. Factors motivating medicalization include, but are not limited to, safety concerns. Involvement of health professionals in advocacy to end FGM/C can address both the supply and demand side of medicalization, but raises ethical concerns regarding dual loyalty. Ongoing debates need to address competing rights claims.

**Summary:**

Polarizing debates have brought little resolution. We call for a focus on common goals of protecting the health and welfare of girls living in communities where FGM/C is upheld and encourage more informed and open dialog.

## Introduction

In December 2012, the United National General Assembly adopted a Resolution to ban female genital mutilations worldwide, “whether committed within or outside a medical institution” [A/RES/67/146]. It reiterated a stance outlined at the first UN-sponsored conference on female genital mutilation/cutting (FGM/C) held in Khartoum over 40 years earlier [1]. While the position of UN agencies has been unambiguous and unwavering over this period [2, 3], proposals and strategies for improving the safety of FGM/C have been forwarded time and time again. In some settings, these strategies have been adopted quietly and privately, whereas in others, proposed policies have attracted the spotlight of media, sparking emotionally charged protests, re-opening longstanding fierce debates, and launching new controversies.

What issues are at stake? What belies the tenacity of these debates?

Female genital mutilation/cutting, also known as female circumcision, encompasses a wide range of procedures involving partial or total removal of the external female genitalia for non-therapeutic reasons. The range of cutting practices has been classified into four types of FGM/C: type I, also known as clitoridectomy, is defined as partial or total removal of the clitoris and/or the prepuce (although it is actually the glans and/or the body of the clitoris that is cut); type II, excision, includes partial or total removal of the clitoris and the labia minora, with or without excision of the labia majora; type III, infibulation, involves narrowing of the vaginal orifice (introitus) with creation of a covering seal by cutting and appositioning or sewing of the labia minora and/or the labia majora, with or without excision of the clitoris; and other forms, include pricking, piercing, incising, scraping, or cauterizing the skin near the clitoris for non-medical reasons, are categorized as type IV [3].

Surveys conducted across parts of Africa, the Middle East, and Southeast Asia document FGM/C prevalence rates ranging from 1% (Uganda, Cameroon) to more than 95% (Guinea and Somalia) [4]. Although survey data are lacking, FGM/C has been described in countries including Colombia, Iran, India, Malaysia, Oman, Pakistan, Russia, and Thailand, as well as in migrant communities throughout the world [4–7]. Worldwide, more than 200 million women and girls are estimated to have undergone some form of FGM/C, while each year, another 3.6 million may be at risk of being cut [8].

Over the last four decades, interventions aimed at ending FGM/C have combined concerted efforts of international organizations, national bodies and governments, as well as religious and civil organizations [7]. Health risk models formed the core of these intervention strategies. Raising awareness about the dangers of FGM/C, experts believed, would spur people to reassess the practice and lead to its abandonment [9, 10]. Early information and education campaigns commonly featured didactic messaging about the short-term, long-term, and obstetrical consequences of FGM/C. Over time, a suite of more comprehensive strategies have emerged. Alternative rite of passage programs, for instance, encourages upholding coming-of-age celebrations but eliminating the cutting aspect of the ritual [11, 12]. Compensate-the cutter programs have retrained traditional circumcisers for different vocations and encouraged them to “drop the knife” [13, 14]. And holistic community empowerment programs are designed to foster community-wide discussions and reappraisal of harmful practices such as FGM/C [15]. Each of these approaches has, to some extent, sought to raise awareness of the health consequences of FGM/C in hope of encouraging abandonment [9, 10]. This may have, however, inadvertently motivated families to turn to the health system in search of safer cutting and encouraged health care providers to comply with their patients’ requests [7].

Analyses of data on FGM/C prevalence from 29 countries (27 African countries plus Yemen and Iraq) showed that in 15 of the 29 countries, there has been little or no change, while 14 nations showed that FGM/C rates are dropping [4]. Change characterized as unacceptably slow has led to calls for intensification of global efforts to end the FGM/C [7, 16, 17]; at the same time, it has prompted some to propose interim strategies to reduce potential harm [18].

## What Is Medicalized FGM/C?

Medicalization is the situation in which health care professionals carry out FGM/C, whether in a health facility or at home or elsewhere, often using surgical tools, anesthetics, and antiseptics in the hope of mitigating immediate complications [7, 19]. This term also applies to performing re-infibulation, re-closure of female external genitalia of women who had been de-infibulated to allow for sexual intercourse, delivery, and/or related gynecologic procedures by doctors or nurse-midwives [19, 20]. It may also include situations in which medical professionals administer painkillers or anesthestics, while cutting is done by the traditional excisor [21]. In countries where health systems are overburdened and experience shortages of health professionals, FGM/C may also be performed by employees who have no formal medical training or clinical knowledge, such as apprentices or community health extension workers [21, 22, 23••]. This pseudo-medicalization can involve the use of surgical tools, pain killers, and antiseptics, and thus may appear to patients to be provided by trained health professionals. Hence, self-reported survey data on medicalized cutting may conflate these two very different groups.

## What Are the Major Patterns and Trends in Medicalization?

A recent overview of data from 25 countries found that rates of medicalization (FGM/C performed by a doctor, nurse, midwife, or other health professional) among women aged 15–49 are highest in five countries: Egypt (38%), Sudan (67%), Guinea (15%), Kenya (15%), and Nigeria (13%) [24••] (Table 1). Elsewhere, medicalized cutting is rare and restricted to geographically defined pockets. All told, the majority of women (74%) report being cut by traditional practitioners. The remaining 26%—totaling nearly 16 million women—reported medicalized cutting [24••].

**Table 1:** Prevalence of FGM/C, medicalization, and total number of women (ages 15–49) cut by health professionals

Country	Data source	Prevalence of FGM/C (%)	Total number of women (15–49)	Rate of medicalization	Total number of women cut	Total cut by health professionals
Benin	DHS 2011–2012	7.3	2,144,241	0.2	156,530	313
Burkina Faso	MICS/DHS 2010	75.8	3,688,866	0.2	2,796,160	5592
Cameroon	DHS 2004	1.4	4,098,869	4.0	57,384	2295
Central African Republic	MICS 2010	24.2	1,172,050	2.3	283,636	6524
Chad	MICS 2010	44.2	2,505,043	6.0	1,107,229	66,434
Côte d’Ivoire	DHS 2012	38.2	5,293,915	0.3	2,022,276	6067
Djibouti	MICS 2006	93.1	193,365	6.0	180,023	10,801
Egypt	DHS 2014	92.3	23,331,079	37.9	21,534,578	8,161,605
Eritrea	DHS 2002	88.7	977,370	0.6	869,559	5219
Gambia	DHS 2013	74.9	486,629	0.3	364,485	1093
Ghana	MICS 2011	3.8	6,041,140	1.2	229,563	2755
Guinea	DHS 2012	96.9	2,518,996	15.4	2,440,907	375,900
Guinea-Bissau	MICS 2014	49.8	381,216	0.2	189,846	380
Iraq	MICS 2011	8.1	7,623,574	6.3	617,509	38,903
Kenya	DHS 2014	21.0	10,877,750	14.7	2,284,328	335,796
Mali	DHS 2012–2013	91.4	3,524,985	0.7	3,221,836	22,553
Mauritania	MICS 2011	69.0	841,940	2.0	580,939	11,619
Niger	DHS 2012	2.0	3,423,589	0.0	68,472	0
Nigeria	DHS 2013	24.8	39,466,768	12.7	9,787,758	1,243,045
Senegal	DHS 2015	24.2	3,529,653	0.0	854,176	0
Sierra Leone	DHS 2013	89.6	1,391,263	1.1	1,246,572	13,712
Sudan	MICS 2014	86.6	8,752,649	66.8	7,579,794	5,063,302
Togo	DHS 2013–2014	3.9	1,618,084	0.0	63,105	0
Tanzania	DHS 2010	14.6	10,156,090	2.3	1,482,789	34,104
Yemen	DHS 2013	18.5	6,040,827	3.0	1,117,553	33,527
Total		26.2			61,137,307	*15,992,493*

Source: [24••]. Note: some more recent surveys do not provide information on medicalization. This table summarizes data from the most recently available survey that included information on type of practitioner of FGM/C

**Fig. 1:**
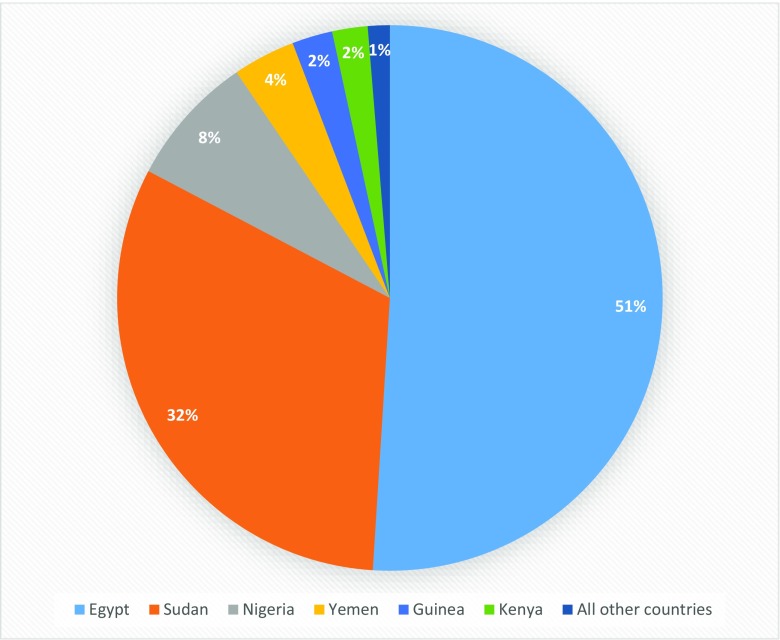
Geographic distribution of women who report having been cut by a medical professional Source: [24••]

Among women exposed to medicalized cutting, 93% live in just three countries: Egypt, Sudan, and Nigeria; more than half (51%) reside in Egypt alone (Fig. 1) [24••]. Notably, these figures do not include Indonesia, a highly populous country where FGM/C is known to be practiced, but where nationally representative data on prevalence of FGM/C among women and rates of medicalization are lacking.

Among daughters, rates of medicalized cutting are substantial (10% or higher) in eight countries: Djibouti, Egypt, Guinea, Iraq, Kenya, Nigeria, Sudan, and Yemen. Mother-daughter comparisons show that medicalization is increasing in each of these countries except Nigeria [24••]. This trend is sharpest in Egypt where, according to the 2014 data, rates have more than doubled between women and daughters (38 and 82%, respectively) [24••]. This increase is likely linked to the fact that in the 1990s, Egypt enacted a policy requiring that FGM/C be performed by a trained medical professional; this policy has now been reversed (for more details, see below and [24••]).

## Medicalization, “Harm Reduction,” and Regulatory Policies

The term “harm reduction” has been used to discuss FGM/C in two distinct ways. First, it has described the desire to reduce the attendant health risks by improving the sanitary conditions under which FGM/C is performed, improving the competency of practitioners, and/or limit the severity of cutting [23••, 25•]. Second, it describes a specific public health strategy known as *harm reduction* that aims to minimize health hazards associated with risky behaviors by encouraging the safest alternative, including, but not limited to, abstinence [26, 27]. This strategy arose in the 1980s to curb the growing HIV/AIDS crisis among IV drug users, and has since informed programs for reducing teen pregnancy, drunk driving, smoking, and various other risky behaviors [27]. Harm reduction recognizes abandonment of or abstaining from a risky behavior as the ideal outcome because it bears lowest risk of harm, but recognizes alternative changes short of abstinence. It promotes the alternative that is culturally or individually acceptable and bears the lowest risk of harm.

Policies regulating the way in which FGM/C is carried out have been described as “temporary transitional measures” for those not yet ready to stop the practice [28–30]. Some have been policies that restrict the type of FGM/C that can be performed, the persons who are permitted to perform the procedure, and/or the setting in which it can be conducted. While the restrictions may have been intended to reduce the risk of more extensive cutting or having FGM/C performed by an unqualified practitioner in an unhygienic setting, not all policies specified a goal of working toward abandonment of FGM/C. For instance, in Kenya in 1931–1932, two Local Native Councils (elected bodies that reported to British colonial administrators) passed resolutions restricting the severity of allowable forms of excision [33]. While these policies prohibited more severe forms of cutting, they did not require circumcisers to encourage abandonment of FGM/C. We suggest that such restrictions be referred to as *regulatory policies* when they do not specifically work toward abandonment of FGM/C. We suggest that the term “harm reduction” should be reserved to describe strategies that seek to minimize medical risk by encouraging the safest acceptable alternative, including abandonment of FGM/C.

## Policies Regulating FGM/C

Efforts to medicalize FGM/C or restrict the severity of cutting have had a long history and have been implemented with varying degrees of success. Colonial governments in Sudan and Kenya instituted regulations on the type of cutting allowed, but were met with strong resistance [31–33]. Similarly, in the 1970s, a recommendation by the Somali Women’s Democratic Organization to replace infibulation with pricking performed in hospitals was not followed [30]. Decades later, Islamic scholars have engaged in debating the religious grounds for different forms of FGM/C [34]. Although FGM/C is not mentioned in the Koran, it is mentioned in some hadiths (recorded sayings and practices of the Prophet Mohamed). One commonly cited hadith attributes the following statement on FGM/C by Prophet Mohamed: “If you cut, do not overdo it, because it brings radiance to the face and is more pleasant for the husband.” Since the authenticity of this hadith is debated, opinions are divided regarding whether FGM/C is permitted or required by Islam [29, 30, 34]. In Indonesia, for instance, even though FGM/C was banned in 2006, two Muslim organizations reportedly continued to condone the practice, advising “not to cut too much” [35]. The Indonesian Ministry of Health subsequently issued a 2011 directive for health professionals to perform scraping on girls [35]. The government, however, repealed this regulation in 2013 and reiterated support for a 2006 law banning FGM/C.

In Egypt, medicalized cutting is carried out primarily by doctors, as opposed to other health workers, and is historically rooted in a 1994 Ministry of Health order requiring state hospitals to set aside 1 day a week for trained physicians to perform FGM/C [36, 37]. In the wake of sharp criticism, this policy was changed, but a loophole allowing for “medically necessary circumcision” was not closed until 2007 [38].

In parallel, in the 1990s as European and North American countries received increased numbers of immigrants from countries where FGM/C is practiced, several proposals were drafted recommending offering pricking in lieu of more severe forms of cutting in children old enough to give consent and for those families where any prevention strategy did not work. While intended to be compromise solutions, balancing respect for cultural values, host countries laws, medical and ethical principles while minimizing health risks [28, 39, 40], and despite having been reviewed (in the Florence case) by a Bioethics Committees that “judged the proposals to be ethical, legal, deontological, efficacious and fair” [40, p. 8], each of these proposals ignited public outrage and were never implemented.

## Stances of Governments and International and Professional Organizations

Numerous medical associations condemn medicalization, including the World Medical Association, the American College of Obstetricians and Gynecologists, the American Medical Association, and the International Federation of Gynecology and Obstetrics [19, 41]. A dissenting view was expressed by the American Association of Pediatrics, whose 2010 policy statement called for allowing pediatricians to perform nicking/pricking [42]. A firestorm of protest led to the subsequent retraction of this statement. Recently, following a 2017 summit in Egypt, public statements opposing medicalization were issued by professional medical associations from Djibouti, Egypt, Somalia, Sudan, and Yemen. Thus, policies by professional organizations around the globe are now aligned with opposition to all forms of medicalization.

On December 20, 2012, the United Nations General Assembly passed a Resolution on Intensifying Global Efforts for the Elimination of Female Genital Mutilation [A/RES/67/146]. Its adoption reflects agreement that FGM/C constitutes a violation of human rights and that all countries should take action to end FGM/C and its medicalization, “take all necessary measures including enacting and enforcing legislation to prohibit FGM” [A/RES/67/146]. Over 40 countries have banned FGM/C by law or constitutional decree. In at least six of these countries (Burkina Faso, Cote d’Ivoire, Egypt, Eritrea, Mauritania, and Senegal), the criminal code specifies an elevated penalty (prison and/or fine) specifically for medical personnel who perform FGM/C, in addition to the possibility of suspending their licenses [43].

## Ongoing Debates Surrounding Medicalization/Harm Reduction

A wide range of debates on medicalization have been critically, and sometimes bitterly, interrogated in a voluminous body of scholarly literature, as well as in the media and political arenas. Many of these debates have centered on three broad concerns: (1) the effects of medicalization on effort to end FGM/C, (2) understanding what drives medicalization and how it can be discouraged, and (3) reconciling competing rights claims surrounding FGM/C.

## Does Medicalization Counteract Efforts to End FGM/C?

At the heart of opposition to medicalization rests a central assumption: that medicalization will counteract efforts to eliminate FGM/C. Medicalization, critics fear, can create the impression that FGM/C can be performed safely and is condoned by respected medical professionals, thus reducing motivation of families to abandon the practice [19, 23••, 44••]. Others have speculated that medicalization or less severe cutting can be an interim step toward abandonment [28–30]. Current evidence is conflicting, a finding that is perhaps not surprising since FGM/C regulations have not been instituted as formal harm reduction policies aimed at encouraging abandonment.

A statistical overview of data from 25 countries showed no association (non-significant correlation) between medicalized FGM/C among daughters and rates of decline in prevalence of FGM/C [24••]. In countries where measurement has been repeated in surveys roughly 5 years apart, substantial changes in both medicalization and prevalence rates are detectable only in Kenya, with FGM/C prevalence rates declining sharply while medicalization remained high. Therefore, at least in this setting, medicalization has not completely counteracted the process of abandonment of FGM/C. By contrast, two countries with the highest rates of medicalization, Egypt and Sudan, show persistently high rates of FGM/C, with change visible only in the youngest age cohorts [24••]. Whether medicalization is hindering this decline is unclear and is best investigated in focused studies.

A recent mixed-methods study (using survey and in-depth interviews) in Egypt reported that doctors did not consistently discourage FGM/C, even when mothers sought advice and expressed concerns for their daughter’s safety [44••]. Another study analyzing the 2008 Egypt DHS data found that households were *less likely* to opt for FGM/C when medicalization was used more widely among their daughter’s peers; the author suggested that medicalization might be associated with changing norms surrounding FGM/C, opening possibilities for abandonment [45]. This interpretation was not supported by a new study in Kenya, where families who adopt medicalized cutting do not consider this to be a step toward abandonment [21]. Further research is needed to reconcile these conflicting findings.

Research on whether medicalization is associated with reduction in severity of cutting also shows mixed results. Studies in Nigeria and Kenya reported that health professionals who performed FGM/C promoted nicking in lieu of clitoridectomy in order to reduce the chance of complications and drawing attention to their practice [46, 47]. Conversely, research in Indonesia documented that while traditional practitioners tended to perform scraping of the clitoris (type IV), health care providers more commonly performed more severe type I FGM/C [48]. Surveys on FGM/C do not always collect information on type of FGM/C, but the limited available data show a shift to less severe nicking/pricking forms of cutting (defined as “cut, no tissue removed”) in countries where medicalization is spreading [24••]. Whether there is any causal role is, however, unclear.

## What Motivates Medicalization of FGM/C and What Can Be Done to Discourage It?

While medicalized FGM/C is found in greatest concentration in three countries (Egypt, Sudan, and Nigeria), health professionals in countries around the world have been approached with requests to cut girls and women [49–53]. These include countries in Africa, Asia, and the Middle East where FGM/C has long been practiced, as well as those that have received migrants, refugees, and asylum seekers from FGM/C practicing communities [19, 54].

The demand for medicalized cutting appears to be driven by a number of factors. First, heightened concerns over potential health complications have motivated parents to seek medicalized and/or less severe cutting [19, 21, 23••, 25•, 44••, 55]. At times, medicalized cutting has been driven by policies restricting traditional practitioners, but allowing health professionals to perform FGM/C (e.g., Egypt in the 1990s, and more recently, Indonesia). Additionally, in some instances, medicalized cutting has been offered as part of routine neonatal care options, even in settings where FGM/C is considered to be a relatively benign procedure [22, 56].

Attention has also focused on understanding the supply side of medicalization. Financial gain is a recurring theme across studies [23••], although it is not always the primary motivating factor. Some health care providers themselves come from FGM/C-practicing families and support its continuation [10, 22, 23••] and are willing to honor the values and wishes of their patients [23••, 25•, 55, 57]. Knowledge of criminal statutes or professional guidelines has been shown to be incomplete [47], and legal bans at times run counter to the professional norms shared by communities of providers [21, 36, 55, 57]. This is particularly evident in setting where health professionals justify performing FGM/C in order to prevent it from being performed by an unskilled practitioner, without pain management, or under unsanitary conditions [23••].

Improving education on FGM/C has become prioritized not only for improving clinical care for affected women, but also preparing health providers to be involved in advocacy. While some countries now require health professionals to educate patients on FGM/C, in others, this duty is considered untenable given the already heavy workloads and time constraints faced by medical providers [10, 58]. Moreover, because few training institutions have comprehensive components on FGM/C in their curricula, health care professionals continue to report having insufficient knowledge on the issue [59].

A number of countries also require health care providers to track and/or prevent FGM/C. An overview of policies in 30 countries (11 countries where FGM/C is practiced and 19 that host migrants from FGM/C-practicing countries) found that 16 countries mandate a “duty to report” cases in which a patient has undergone FGM/C, while 18 countries require formal action to be taken when health professionals suspect that FGM/C will be carried (duty to avert) [58]. These reporting requirements raise the ethical dilemma of dual loyalty, where health care providers have an obligation to protect the confidentiality of their patients at the same time as having a duty to report to the state [60]. Whether mandated actions to prevent FGM/C on a girl or her siblings are offset by concerns regarding violating confidentiality, eroding trust between providers and their patients, and setting off protection orders that remove a girl from her home and/or lead to the imprisonment of her parents are matters of grave concern [54, 61].

Those calling for stricter enforcement of bans on medicalized FGM/C have encouraged sanctioning health care institutions that do not enforce such policies and criminally prosecuting violators. Legal charges have been issued against medical professionals accused of performing FGM/C in countries including Egypt [62], the UK [63], and the USA [64]. Complexities in these cases include determining whether legal restrictions apply to nicking, pricking or scraping, and determining when FGM/C is “medically necessary.” Additionally, the extent to which high-profile cases deter other medical professionals from performing FGM/C or drive the practice underground is poorly understood.

## Should There Be Zero Tolerance of All Forms of FGM/C and Medicalization?

Ongoing debates have addressed how to distinguish acceptable risk from intolerable harm, or who has the right to make such distinctions. These are interpretive issues, linked to legal, ethical, medical, and human rights claims about the limits of individual autonomy and tolerance of multiculturalism.

The Platform for Action developed at the 1995 Fourth World Conference on Women laid a blueprint for framing FGM/C as a human rights violation [65]. Drawing on these principles, the UN advanced a “zero tolerance” approach for opposing all forms of FGM/C, a position that reflected a break from the earlier health framework and vexing questions it spurred about whether or how health risks might be minimized [66]. Strategies to promote and protect these rights have faced the challenge of simultaneously addressing competing rights claims: how can rights of the child, women’s rights to freedom from discrimination, freedom from torture, and the right to bodily integrity and health be reconciled with a right to culture or religious freedom [66–68]?

The strictest application of the zero tolerance stance prohibits any non-therapeutic procedure involving the female genitalia. However, when prohibition is linked to the concept of harm, as is stipulated in certain criminal codes [43], questions arise as to whether restrictions also apply to type IV procedures (nicking, pricking, or scraping of the clitoris or clitoral hood) that do not produce anatomical changes [69]. In the mid-1990s in the Netherlands, a recommendation called for differentiating “tissue impairing and non-tissue impairing circumcision and non-mutilating ritual incision,” and suggested that doctors be allowed to perform an anesthetized prick of the clitoral covering [28] (p. 285). Similar proposals were also considered in Seattle, Washington [30], and Florence, Italy [40]. Two decades later, medical ethicists endorsed “de minimis” forms of FGM/C, such as pricking, that were said to bear no long-term medical risks [18]. Critics question the veracity of the claim that the long-term consequences are rare [70], as well as the merits of using harm as grounds for defining acceptable versus unacceptable forms of FGM/C. Irrespective of harm, critics charge, FGM/C is rooted in the gender discrimination and therefore violates the fundamental human rights of girls and women [19].

Debates have also lingered over unresolved questions on consent. Social pressures in societies where FGM/C is widely practiced were argued to limit women’s ability to provide meaningful consent [71, 72] and justify protectionist measures. Critics, however, questioned whether such measures compound restrictions on women’s autonomy, possibly advancing an image of women as victims who are incapable of reasoned decision-making regarding acceptable risk [73, 74]. Moreover, perplexing contradictions arise from banning FGM/C while upholding permissive standards regarding female genital cosmetic surgeries, some of which bear striking similarity to certain form of FGM/C (labia reduction, clitoral reductions, and a form of labial adhesion known as “The Barbie” [72, 75, 76•]. While this concern first arose in Europe and North America, it now extends to Egypt, where some doctors who perform FGM/C have adopted a discursive change, calling it a “cosmetic procedure” [57].

In the case of minors, the issue of consent regarding FGM/C is tied parental authority. Because children below a certain age have “diminished capacity” to make medical decisions [77], the “best interest” principle grants parents (or legal guardians) latitude to make decisions on a child’s behalf [78]. However, the state at times intervenes, such as when parents have refused medical treatment for life threatening conditions on the basis of religious beliefs [78]. For FGM/C, the calculus of acceptable risk rests on weighing competing rights enshrined in the Convention on the Rights of the Child: a child’s right to practice her culture or religion (Article 23), and a child’s right to health (Article 30). The Joint UN Policy Statement on FGM/C recognizes that parents “who take the decision to submit their daughter to female genital mutilation perceive the benefits to be gained from this procedure to be outweighed by the risks involved” [3] (p. 9). Yet, it firmly concludes that “this perception cannot justify a permanent and potentially life-changing practice that constitutes a violation of a girls’ fundamental human rights” [3] (p. 9). This position is endorsed in criminal statutes that restrict not only type III, but also types I and II among minors. However, where statutes pose restrictions based on harm, rather than zero tolerance, the legal status of nicking, pricking, piercing, and scraping (type IV) are less clear [43].

A parallel debate questions whether double-standards are being used to oppose type I and type IV (nicking, pricking, and scraping, in particular) FGM/C while condoning routine male neonatal circumcision. Commentators calling for equal treatment differ in terms of concluding whether both male and female genital cutting on minors should be allowed or prohibited [79–83]. Earp has argued that all forms of genital cutting on minors, regardless of health consequences, may form an “intimate violation” [84••], and he instead advances the idea of “genital autonomy,” wherein decisions are delayed until children reach an age at which they can provide valid consent [85]. This position, however, sets decision-making regarding FGM/C apart from other widely accepted domains of parental decision-making authority that may influence a child’s mental, moral, and spiritual development [68]. However, Earp’s view aligns with a 2012 opinion of a Higher Regional Court in Cologne, Germany that classified circumcision of boys as “bodily harm” and ruled that the “fundamental rights of the child to bodily integrity outweigh the fundamental rights of the parents” [86]. Vocal protests from Jewish and Muslim groups characterized the Court’s opinion as “an unprecedented intrusion of the right to self-determination of religious communities” [87], and prompted the German government to pass legislation allowing ritual male circumcision when performed by a medical professional [88]. An upcoming criminal trial in the USA may soon be asked to render a similar decision in a case on FGM/C [64].

## Conclusion

While controversies have long surrounded the issue of FGM/C, some of the most vexing and tenacious debates have centered on its medicalization and regulations on the severity of cutting. The intractability of these debates rest, in part, upon the tendency to conflate moral and medical arguments [84••], as well as the slow generation of sound scientific data to test empirical claims [19, 66, 89, 90]. Recent decades have witnessed a dramatic increase in scientific evidence on health risks associated with FGM/C and behavior change. Yet, there are still gaps in knowledge, particularly on risks associated with type I and type IV forms of FGM/C, and there remains a pressing need to reconcile the evidence base with ethical, moral, and legal standards.

Growing consensus on defining FGM/C as a human rights violation underscores that concerns are not limited to minimizing health risks, but rather extend to broader concerns on child protection and well-being, consent, bodily integrity, and discrimination against women. Medical ethicists, legal experts, and policymakers alike have been forced to confront competing rights claims, including the right to health, right to bodily integrity, rights of the child, right to culture, and right to religious freedom. The lack of clear cut, definitive answers regarding the priority of competing claims have given fuel to ongoing debates surrounding medicalization, some of which have now become objects of scrutiny in courts of law around the world.

Polarizing debates have cast each side in a negative light. Are proposals for less extensive or medicalized cutting condoning child abuse and willing to undercut efforts to end FGM/C, or are they compassionate, pragmatic compromise solutions intended to protect the health of girls? Are zero tolerance platforms safeguarding the human rights of girls, or staunchly defending a moral high ground at any cost? We suggest that these extreme positions deflect focus from the common ground. The central interest of all parties in these debates, we believe, is to seek solutions to best protect the health and welfare of girls living in communities where FGM/C practices are upheld. Resolution may require calling for more informed and open dialog to better understand and address the constraints on bringing a rapid end to FGM/C, and the effects of changes in type of cutting or choice of providers that are already underway in parts of the world. While legal action serves to impose regulatory restriction and punish violators, it is unclear if this will discourage the supply and demand for medicalization, or drive the practice underground. It also remains questionable as to whether nuanced understandings and solutions will arise in the adversarial context of courts of law. Hence, the best chances for achieving lasting resolution will likely involve continued dialog between patients, providers, and all of those working ardently to change norms and protect the rights and well-being of girls and women.
